# Adaptation of the Good Spirit, Good Life quality of life tool for remote Indigenous Australians

**DOI:** 10.1007/s11136-025-04083-x

**Published:** 2025-10-26

**Authors:** Lianne Gilchrist, Leon Flicker, Dawn Bessarab, Roslyn Malay, Laurie Yambo, Betty Sagigi, Chenoa Wapau, Sarah Russell, Rachel Quigley, Caleb Rivers, Zoë Hyde, Esther Chaney, Edward Strivens, Christianne White, Kate Smith

**Affiliations:** 1 Good Spirit Good Life Centre of Research Excellence, Centre for Aboriginal Medical and Dental Health, Medical School, University of Western Australia, Perth, Australia; 2 Centre for Aboriginal Medical and Dental Health, Medical School, University of Western Australia, Perth, Australia; 3 Western Australian Centre for Health and Ageing, Medical School, University of Western Australia, Perth, Australia; 4 Queensland Health, Torres and Cape Hospital and Health Service, Thursday Island, Australia; 5 College of Medicine and Dentistry, James Cook University, Cairns, Australia; 6 Cairns and Hinterland Hospital and Health Service, Queensland Health, Cairns, Australia

**Keywords:** Indigenous, Assessment, Translation, Wellbeing, Adaptation, Ageing

## Abstract

**Purpose:**

The Good Spirit, Good Life (GSGL) assessment tool was co-developed in urban and regional Australia to address quality of life (QoL) for older Aboriginal and Torres Strait Islander peoples and inform culturally responsive care. This study aimed to determine the acceptability and validity of the GSGL tool in Australian remote settings.

**Methods:**

A co-design methodology was applied to this study. Yarning groups were conducted in 5 communities across 2 remote regions of Australia with older Aboriginal and Torres Strait Islander people. Required adaptations to the tool were refined with governance groups in each region. Forward and back translation was performed for the adapted tool with consensus achieved through an expert committee.

**Results:**

Adaptations to the GSGL tool involved small wording changes to two items (Country/Island Home and Elder role). Five items were adapted through additional prompts and examples (culture, respect, supports and services, safety and security, basic needs). The remaining five items were retained (family and friends, community, health, spirituality, future planning). During forward and back translation, translation errors were identified with an expert language committee highlighting the importance of clear translation methods.

**Conclusion:**

The adapted GSGL tool is an acceptable QoL tool for use in health and aged care with urban, regional and remote-living Aboriginal and Torres Strait Islander Australians. When translating a tool, forward-back translation with an expert language committee is recommended to reach concordance in meaning. The adapted GSGL tool is suitable for use with an interpreter when required.

**Supplementary Information:**

The online version contains supplementary material available at 10.1007/s11136-025-04083-x.

## Introduction

Adaptation of assessment tools can be a cost and resource effective means of expanding the applicability of an instrument that has existing reliability and validity to other populations [1–3]. A key objective of cross-cultural adaptation of an assessment measure is to ensure equivalence between source and target versions of the instrument [4]. This requires not only linguistic translation, but also cultural adaptations to ensure content validity of the assessment tool between different cultures [4]. The adaptation process generally encompasses:

“deciding whether or not a test in a second language and culture could measure the same construct in the first language; selecting translators; choosing a design for evaluating the work of test translators (e.g. forward and backward translations); choosing any necessary accommodations; modifying the test format; conducting the translation; checking the equivalence of the test in the second language and culture and conducting other necessary validity studies” [5, p. 6].

There is no clear consensus on the most effective methodological approaches for instrument adaptation [6–8]. Similarly, there is no established best practice for ensuring equivalence in instrument translation presented in the literature [9]. In their review, Epstein et al. [6] reported a lack of evidence supporting one methodological approach over another, concluding that many methods achieve comparable results. They proposed that choosing a suitable adaptation method should be determined by the context in which the instrument will be used. This may be influenced by factors such as the research objectives, translator availability, budget and time constraints and the researcher’s epistemological perspective [10, 11].

This paper outlines the methodology and methods used in adapting the Good Spirit, Good Life (GSGL) assessment tool for use in remote-living Aboriginal and Torres Strait Islander populations. The GSGL is the first quality of life (QoL) tool developed for older Aboriginal and Torres Strait Islander people, who are the First Peoples of Australia. The GSGL assessment tool and framework were co-designed with older Aboriginal people to centre the Aboriginal and Torres Strait Islander worldview of having a good life [12, 13]. The GSGL tool is validated for use with older Aboriginal people from urban or regional areas. Adaptations to ensure acceptability and validity of the GSGL tool in remote regions and for use in Torres Strait communities may be required. Acceptability in the context of this paper, refers to how well received the GSGL tool is amongst older remote-living Aboriginal and Torres Strait Islander populations [14]. Validity refers to the degree to which the items of the tool are comprehensive, relevant and satisfactory to the target population [15].

### Extent

**Fig. 1:**
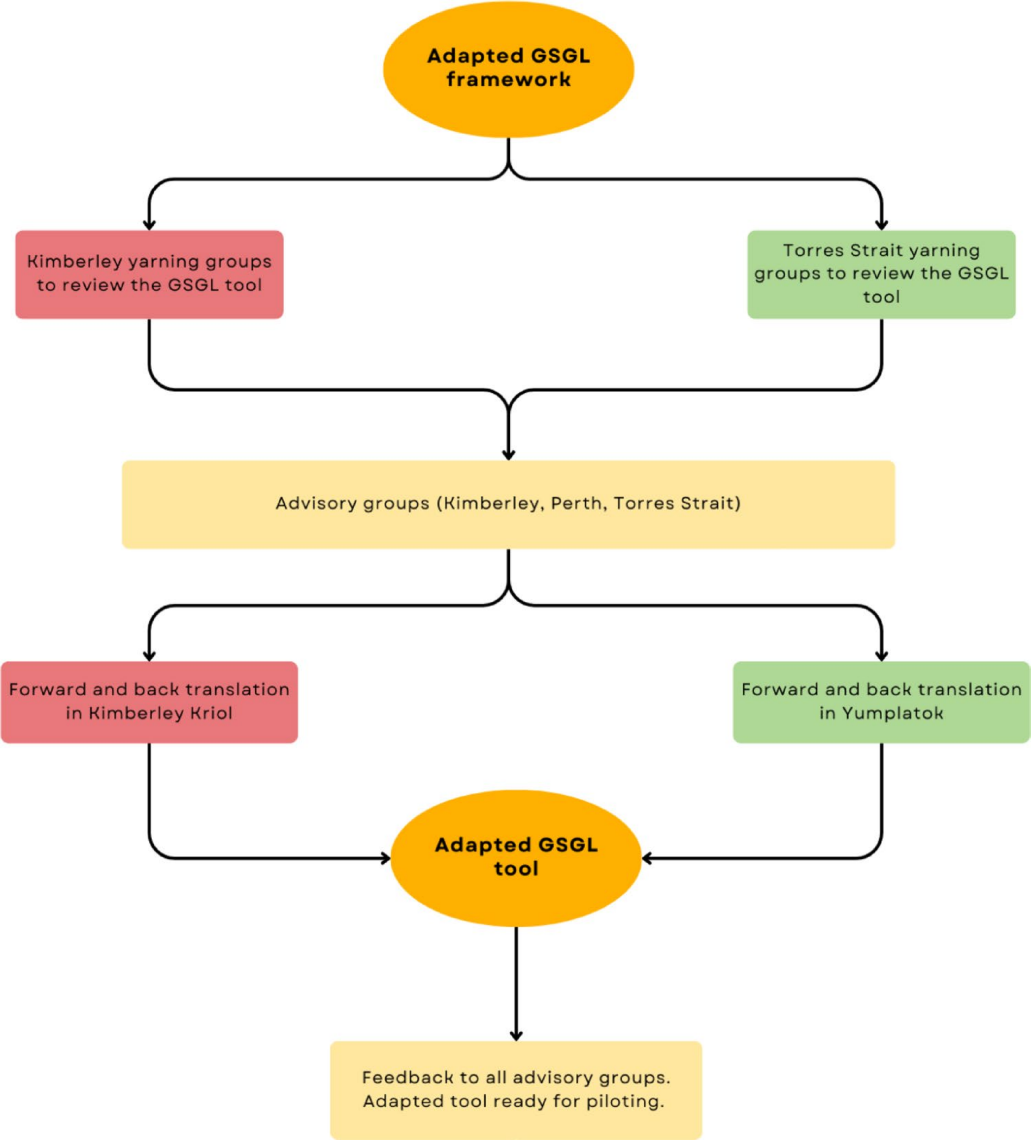
Study flow diagram

This research, co-designed with older Aboriginal and Torres Strait Islander peoples, was conducted in 4 stages: (i) community consultation and support for the study; (ii) review and adaptation of the GSGL framework; (iii) review and adaptation of the GSGL assessment tool; and (iv) forward and back translation. Stages 1–2 have already been completed [16]. This study focuses on stages 3–4 (Fig. 1). The aim of this study is to explore the acceptability of the urban/regional GSGL tool for use in older remote Aboriginal and Torres Strait Islander populations. Given that a small adaptation to the GSGL framework was required in stage 2 of this study (with the addition of Island Home to the Country domain) [16], it was hypothesised that the GSGL tool may also require adaptation to ensure acceptability for remote-living older Aboriginal and Torres Strait Islander populations.

## Methods

### Methodologies

Indigenous Research Methodologies were applied including co-design, Indigenous-led and culturally governed research, culturally centred methods including yarning, and the focus on a culturally informed and co-designed tool [17, 18]. These decolonising methodologies privileged Indigenous perspectives and ways of knowing, being and doing, promoting cultural safety and ensuring that research outcomes reflected the goals and needs of the communities involved [19].

### Setting

This study was conducted in two remote regions, the West Kimberley region of Western Australia (WA) and the Torres Strait Islands and Northern Peninsula Area (NPA) of Queensland (Qld), Australia. There were 2 participating communities in the Kimberley region (Derby and Mowanjum) and 3 communities in the Torres Strait region (Thursday Island, Horn Island and New Mapoon).

### Advisory groups

Three advisory groups were involved in this research: the Kimberley Elders Advisory Group (Derby, WA); the Knowledge Circle (Thursday Island, Qld); and the Perth Elders Governance Group (Perth, WA). These groups reviewed and approved research findings, and provided cultural oversight for the regions in which they were located. The Perth Elders Governance Group were involved in the original development and validation of the GSGL assessment package and have maintained ongoing involvement in all GSGL-related research. Data from the yarning groups, GSGL adaptations, and final results from translation work were discussed with all advisory groups. The research process for this study is outlined in Fig. 1.

### Participants

A purposive sampling strategy was employed to achieve a broad representation of participant ages, sex, language groups and service use. Locally based Aboriginal and Torres Strait Islander researchers and representatives from community health and aged care services assisted with recruitment (identifying potential participants who met the inclusion criteria) and provided guidance to ensure respect and adherence to cultural values and protocols.

The inclusion criteria for this study were: (i) identifying as Aboriginal and/or Torres Strait Islander; (ii) aged ≥ 45 years; (iii) living in a remote region of Australia. People with high care needs were excluded unless a carer was present to support participation in the study. Where possible, participants were invited to be involved in all stages of the study, although this was not a requirement. All participants provided written informed consent.

### Yarning groups

Initial data were obtained through 6 yarning groups with data saturation reached within each group once discussions revealed no further new information. Each yarning group built iteratively on insights from previous group discussions until data saturation was reached [20]. Aboriginal and Torres Strait Islander peoples use yarning as a “a conversational process that involves the telling and sharing of stories and information” [21]. Yarning is recognised as a culturally grounded research method and culturally safe approach to engaging Aboriginal and Torres Strait Islander people in research by privileging Indigenous ways of knowing, being and doing. It is a familiar and meaningful communication method for Aboriginal and Torres Strait Islander peoples and is co-operative, relational and embraces cultural protocols. Yarning groups extend this approach to a collective setting, where participants share stories and experiences in a culturally safe and respectful environment, guided by Indigenous ways of knowing [22, 23].

Centring Indigenous research methodologies, local Aboriginal and Torres Strait Islander researchers in the Kimberley and Torres Strait undertook data collection and analysis. Data collection through yarning groups was conducted by Aboriginal and Torres Strait Islander authors Gilchrist, Malay, Wapau, Sagigi, and Yambo, and non-Indigenous authors Quigley, Russell and White. Drawing on the findings of our previous work, which necessitated a small wording change to the GSGL framework [16], yarning groups in the present study explored the acceptability of the 12 items in the GSGL assessment tool, through (i) feedback on the clarity and relatability of the tool items, and (ii) suggestions for changes to the tool items to improve clarity and relatability. A copy of the GSGL tool was provided to each participant, and researchers leading these yarning groups facilitated discussions for each of the 12 items in the tool. Data were captured using electronic audio recorders and handwritten fieldnotes.

### Analysis

Audio recordings were transcribed and entered into NVivo software version 14 for analysis (QSR International, Burlington, Massachusetts). Deductive thematic analysis was employed using pre-determined codes based on the 12 item areas in the GSGL tool. Data analysis was completed by Aboriginal and Torres Strait Islander researchers (Gilchrist, Rivers, Wapau) and non-Indigenous researcher (Chaney).

**Fig. 2:**
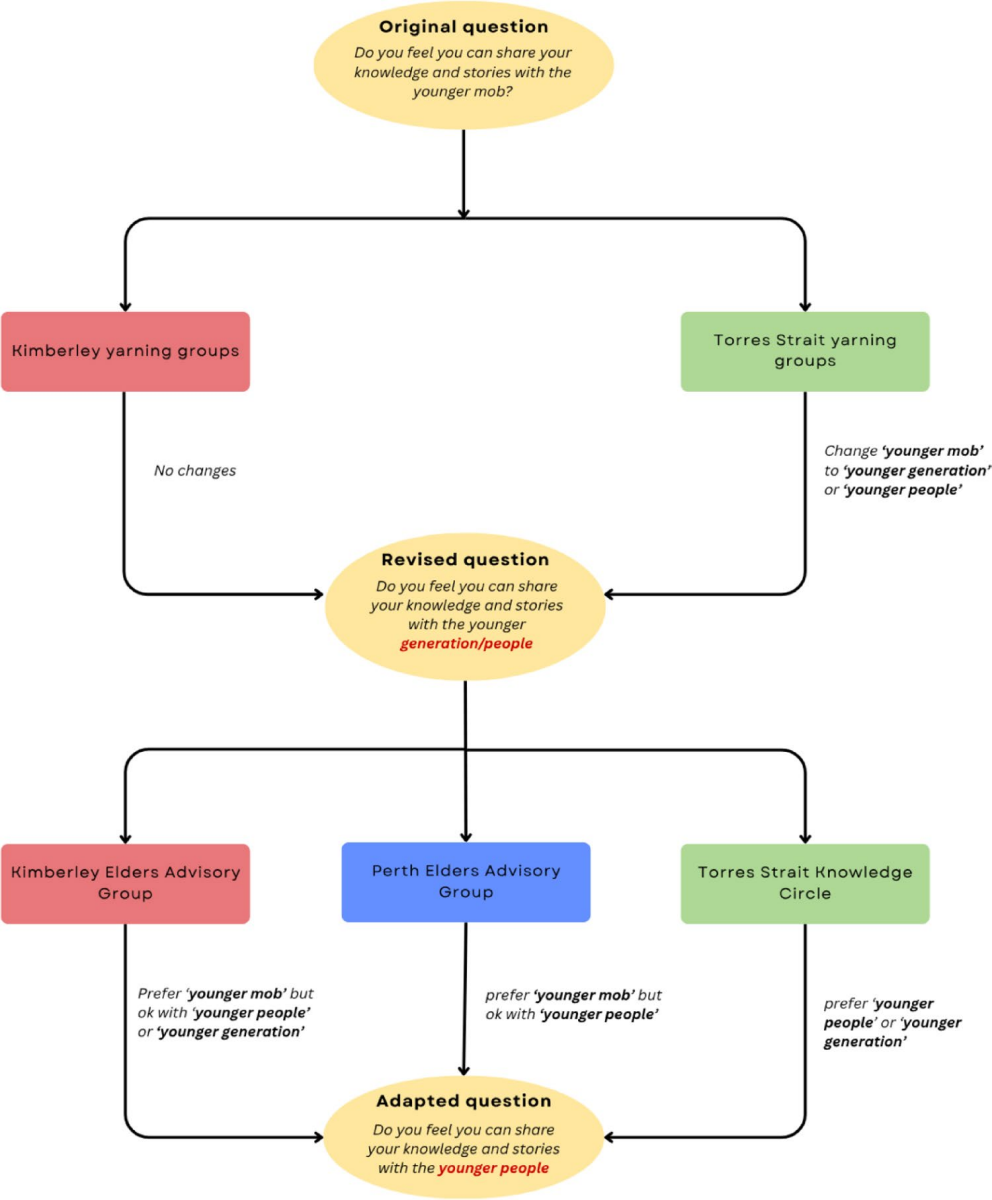
The iterative process for the Good Spirit Good Life tool adaptation

Yarning group data on suggested changes to the wording of the GSGL items were collated into a single document and classified according to the following categories: (i) no changes; (ii) wording changes; (iii) prompt and example changes; and (iv) general feedback. These were subsequently colour coded to visually qualify the types of changes suggested. This assisted in presenting and discussing the findings with fellow researchers, workshopping possible adaptations to questions and refining these with the advisory groups, who were consulted separately. Using an iterative process, each advisory group was updated on feedback from the other advisory groups to inform discussions. The key objective of these discussions was to ensure the tool was both inclusive of the yarning group feedback and representative of older Aboriginal and Torres Strait Islander peoples’ perspectives across diverse regions. This iterative process is outlined in Fig. 2.

### Translation

Once adaptations were agreed upon by all advisory groups, the GSGL tool was further tested through forward-back translation methods to determine suitability for use with interpreters. This was to ensure the essential meaning of the assessment items would not change when interpreted [4].

Forward-back translation was completed in the two dominant languages spoken in each region, Kimberley Kriol and Yumplatok (Torres Strait Creole), as endorsed by the advisory groups. Forward translation was conducted by an Aboriginal and/or Torres Strait Islander translator with a professional interpreting and translation service. This involved converting the GSGL assessment tool from its original standard English version into the recommended language in each region. Back translation involved converting the translated version of the tool back into English, thus revealing discrepancies between the source and target versions [6]. Aboriginal Interpreting Western Australia Aboriginal Corporation (AIWAAC) interpreters completed the translation component of this study in Kimberley Kriol. Ethnolink Translation Services interpreters completed the translation into Yumplatok. A guide was provided by the research team to ensure consistency in the translation methods across both services (Appendix A).

The tool was divided into 14 segments: (i) instructions to the participant (segment 1); (ii) the 12 items/questions (segment 2–13); and (iii) the response key (segment 14). The guidelines outlined by Beaton et al. [4] were used to iteratively inform the translation stage of this study, specifically following stages 1–4 (initial translation, synthesis of translations, back translations and expert committee). These quality assurance methods, and any related issues or comments, were documented in writing to capture the complete process and to assist with further analysis. Analysis was completed by an expert committee which was established for the study, comprising the research team (including multilingual researchers and source instrument developers), translators and linguists.

### Ethics

Approval was granted by the Western Australian Aboriginal Health Ethics Committee (HREC1072), the University of Western Australia Human Research Ethics Committee (reciprocal approval HREC1072), the Far North Queensland Human Research Ethics Committee (HREC/2020/QCH/59342 − 1406) and James Cook University Human Research Ethics Committee (H8063).

## Results

Twenty-eight participants aged 49–83 years were recruited for this study. Eighteen participants were female (64%) and 10 participants were male (36%). Eight participants (29%) were receiving aged care services. Seventeen participants were recruited in the Kimberley region of Western Australia, representing 11 language groups. Eleven participants were recruited in the Torres Strait and NPA of Queensland, representing 6 island and 3 mainland communities.

### Yarning groups

Participants unanimously agreed that the original wording of the ‘family and friends’ and ‘spirituality’ items were acceptable with no further changes suggested. In the Kimberley region, two yarning groups found the wording of the ‘Country’ item acceptable. Participants in the Mowanjum women’s yarning group suggested changing the word “connecting” in the ‘Country’ item, although alternative wording was not stated. All yarning groups in the Torres Strait region reported that the question should include the term ‘Island Home’ in the ‘Country’ item.

*So might be Country/Island Home… The person who’s interviewing the elderly person has to know the background of them*.

(New Mapoon participant)

Five of the six yarning groups suggested that the wording of the ‘community’ item should be “your community” rather than “the Aboriginal and/or Torres Strait Islander community”. The remaining yarning group found the original wording of the question acceptable. The wording of the ‘culture’ item was acceptable to all yarning groups in the Kimberley. In the Torres Strait region, further examples were suggested to improve the clarity of the question.

*When they have cultural events at school*,* they do dancing*,* they do weaving*,* they do… whatever tradition things we do. We pass it onto our children.*

(Horn Island participant)

No changes were suggested to the wording of the ‘health’ item in three of the yarning groups (Derby, Mowanjum men and Thursday Island). General feedback was provided by New Mapoon participants to expand the idea of health to include “feeling good” or “feeling well”. Participants from two yarning groups suggested changes to the wording of the question. These were:

*Are you alright with your health?*

(Mowanjum women’s group participant)

*Do you do things to take care of your health and wellbeing?*

(Horn Island participant)

Three yarning groups felt that no changes were required for the ‘respect’ item. Mowanjum women and Thursday Island participants suggested changing the wording to include respect from others. Examples were suggested from Horn Island participants to enhance the item, such as ‘being addressed respectfully’. The ‘Elder role’ item was acceptable to all Kimberley yarning groups. Participants in the Torres Strait region indicated that the word ‘mob’ was not commonly used by older Torres Strait Islander peoples. The terms ‘people’ and ‘generation’ were suggested instead.

No changes to the ‘supports and services’ item were suggested by the New Mapoon participants. The Derby yarning group gave feedback sharing frequent experiences of culturally unsafe services and pervasive racism. The Horn Island yarning group suggested the alternative wording *“Do supports and services listen to you?”.* The remaining yarning groups suggested wording changes and/or examples that included information referring to the person’s home and community.

*Do you feel safe in your home, do you feel safe in your community?*

(Mowanjum men’s group participant)

Participants in Derby and New Mapoon found the ‘future planning’ item acceptable. However, there was general feedback from the remaining yarning groups to broach this topic with sensitivity. There were some suggestions for additional examples (last wishes, advance care plans, wills) and one suggestion for a small wording change.

*Do you have a plan in place for your future?*

(Thursday Island participant)

Three yarning groups found the ‘basic needs’ item acceptable with no changes required. One yarning group suggested using different examples (bills, ‘basic card’ and fuel). Participants from another yarning group were concerned about the question being intrusive and advised sensitivity when discussing the topic. One yarning group suggested a wording change.

*Can you afford food, housing and clothing?*

(Thursday Island participant)

### Advisory groups

#### Retained items

The ‘family and friends’ and ‘spirituality’ items were unanimously accepted by participants; these questions were retained without further refinement. Three other items were retained although further discussions with the advisory groups were required to finalise these decisions. For the ‘community’ item, although a small wording change was suggested by several yarning groups, following discussions with the advisory groups, a final decision was made to retain the original wording to allow for broader scope in the question. For the ‘health’ item, a further prompt of ‘mind, body and spirit’ was discussed to capture the holistic components of health. However, consensus for this change from the advisory groups was not achieved, so the original question was retained. Despite suggestions for additional examples given by some yarning groups, the ‘future planning’ item was retained, as these suggestions were not considered to significantly enhance the item. However, the suggestion to approach this topic sensitively was agreed upon by all advisory groups. Additional information was added to the GSGL instruction booklet accordingly.

#### Changes to items

Items requiring small wording changes were ‘Country/Island Home’ and ‘Elder role’. The ‘Country/Island Home item was updated to reflect the change accepted in the GSGL framework [16]. As vernacular used to refer to ancestral lands differs between Torres Strait Islander and Aboriginal communities, the item was replaced with two versions of the same item; one for Aboriginal people and one for Torres Strait Islanders. The ‘Elder role’ item was also changed to reflect vernacular differences between Aboriginal and Torres Strait Islander peoples. The term ‘younger people’ was agreed upon by all advisory groups as an acceptable replacement to the term ‘younger mob’. Although the original wording was preferred by the Kimberley and Perth advisory groups, the importance of ensuring that the language in the tool was widely acceptable was prioritised.

#### Changes to examples and prompts

Five items were adapted to include additional prompts and examples to encompass remote Aboriginal and Torres Strait Islander perspectives. For the ‘culture’ item, the example of ‘Aboriginal events and meetings’ was broadened to ‘cultural events and meetings’ to be inclusive of both Aboriginal and Torres Strait Islander cultures. The additional examples from participants of ‘painting, weaving, dancing and sorry time’ were agreed with by advisory groups. For the ‘respect’ item, the additional example of “being listened to respectfully” was considered to capture all feedback from the yarning groups without the need to change any wording in the main question. The examples of “your health/community/aged care service” were added to the ‘supports and services’ item to provide further clarity in the question if needed. Similarly, the ‘safety and security’ item was refined to include the example of “in your home/aged care/community”. The examples in the ‘basic needs’ item were adapted to include “bills, transport and medication”. All changes to examples and prompts had the advisory groups’ approval. The iterative process of adapting the GSGL items with the yarning group and advisory groups is included in Appendix B. The final adapted GSGL tool is shown in Appendix C.

### Translations

**Fig. 3:**
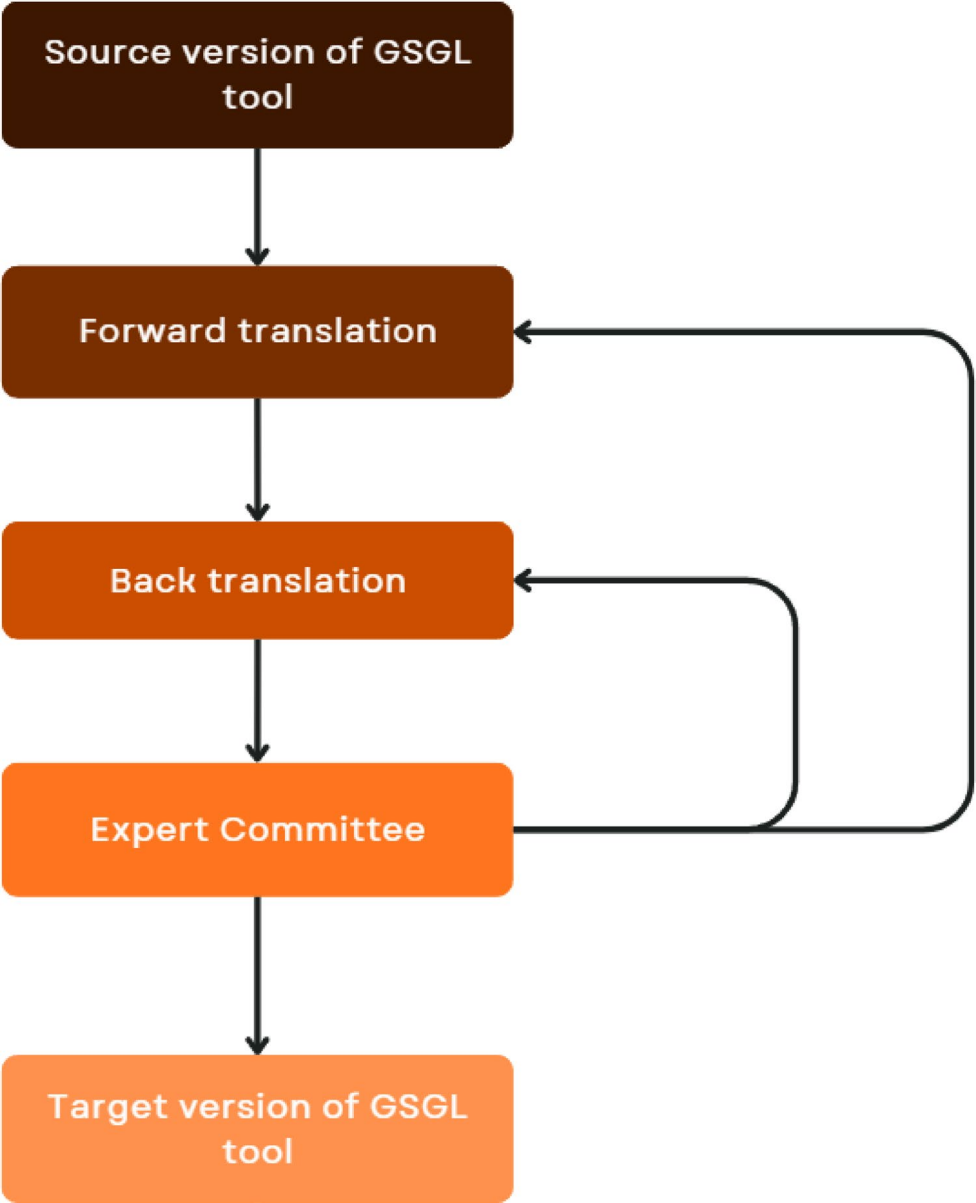
Translation process for adapting the GSGL tool

The following results detail the translation stage of tool refinement (Fig. 3). This process was completed separately for Kimberley Kriol and Yumplatok.

#### Translation results for Kimberley kriol

The expert committee determined that two segments required further revisions following the initial forward and backwards translation process. These were the ‘respect’ item and the response categories. The first back translation for the ‘respect’ item was “do other people respect Elders?”. As this was considered semantically different from the item in English, a second forward translation was required. Equivalence was then determined by the expert committee through the second back translation. In the response categories, the forward translator could not find the Kriol words for ‘most of the time’. This prompted a second forward-back translation to be completed, which was then determined to be equivalent. The original response categories of ‘all the time’, ‘most of the time’, ‘sometimes’, ‘not much’ and ‘never’, were therefore retained in the tool.

#### Translation results for Yumplatok

The expert committee determined that two main segments required further revisions. Translation errors were detected in the back translation for both the instructions and the ‘future planning’ item. For the instructions, the Yumplatok word for ‘life’ was translated to ‘laugh/laughter’. These words were initially identified by the translation service as homophones (sounding the same but having different meanings). However, a researcher and native speaker of Yumplatok on the expert committee indicated that these words are not homophones, although the words may sound similar. This was verified by a second Yumplatok native speaker and researcher. A repeat forward and back translation of the instructions revealed no issue. For the ‘future planning’ question, the review of the back translation revealed a translation error where the phrase ‘do you’ was back translated to ‘did I’ resulting in a semantic change to the item. Again, this was identified by Yumplatok speaking researchers on the expert committee. A second forward and back translation revealed no issues in the translation and was determined to be semantically equivalent.

## Discussion

The GSGL tool was found to be acceptable for older remote-living Aboriginal and Torres Strait Islander peoples with some adaptations. Adaptations involved minor wording changes to two items (Country/Island Home and Elder role) and additional prompts and examples for five items (culture, respect, supports and services, safety and security, basic needs). The original wording and examples were retained for the remaining five items (family and friends, community, health, spirituality, future planning).

The decision to retain the original wording of the ‘community’ item was made to encompass the wide meanings and experiences of community for Aboriginal and Torres Strait Islander peoples. Participants described community as belonging to land, family, and cultural/language groups, with membership into a new community group articulated as a gradual process of earning trust, respect, and acceptance. For Aboriginal and Torres Strait Islander peoples, community is about identifying and belonging to a group of Aboriginal and Torres Strait Islander people which can be expressed in different ways. Dudgeon et al. [24, p. 16] described contemporary Aboriginal [and Torres Strait Islander] communities as “dynamic and flexible, including many networks, politics, (set and shifting) affiliations, and other layers of significance”. For example, a person may have close connections with their community through family networks, and a broader connection to the Aboriginal and Torres Strait Islander community at large through participation in events and activities. It is also important to recognise the impact of past government policies and how these have shaped experiences and perspectives of belonging to community. Of particular relevance is ensuring the GSGL is trauma-informed for members of the Stolen Generation (Aboriginal and Torres Strait Islander peoples who were forcibly removed from family as children). To best capture the diversity and complexity of the concept of community, the original wording of the item was retained.

Participants provided mixed responses regarding the acceptability of the ‘health’ item prompting further examination with advisory groups. Although the prompt “mind, body and spirit” was considered, consensus was not reached with advisory groups. Additionally, concerns regarding spirit being the central concept of the tool and important to each item led to the exclusion of this suggested prompt. Suggestions from the yarning groups for the ‘future planning’ item included honouring last wishes, completing wills and advance care plans. However, the item was retained to ensure the scope remained broad and did not disproportionately focus on end-of-life decisions. Future planning can be viewed from a perspective of ageing well, encompassing aspects of health, care and supports, family and any other plans for the future [25].

The adaptation to the ‘Country/Island Home’ item directly relates to the domain change in the GSGL framework detailed in our previous study [16]. The question for this item is now presented as two options, requiring the assessor to determine whether Island Home or Country would be most appropriate via a social yarn prior to assessment [22]. This aims to limit confusion when asking the older person about their connection to their ancestral lands and to increase the cultural sensitivity of the GSGL tool. There was a single word change in the ‘Elder role’ item reflecting both Aboriginal and Torres Strait Islander vernacular. Although the word “mob” is accepted in the Aboriginal community, it was agreed that the alternative word, “people”, would be widely acceptable for both Aboriginal and Torres Strait Islander peoples. Through the refinement process, it was determined that the cultural relevance of many of the questions could be enhanced by providing additional prompts and examples, which were included to encompass remote Aboriginal and Torres Strait Islander perspectives and improve clarity.

In the absence of empirical evidence to support the use of one translation methodology over another, Epstein et al. [2] noted that many studies have recommended the use of an expert committee, focus groups and back translation. This study has incorporated these key components. Specifically, methodological guidelines of Beaton et al., [4], which have been widely replicated in the literature, have been used to inform this study [3]. Forward and back translation was completed with an expert committee pinpointing the source and consequence of errors identified. To limit the risk of bias in this process, forward and back translation was repeated, to ensure sources of variance had been resolved [8]. This has contributed to a robust methodology and confidence that the adapted GSGL tool has maintained equivalence through the translation process.

This study was conducted using co-design methods through strong collaboration with Aboriginal and Torres Strait Islander community participants, advisory group members, researchers, and an expert committee. These methods have ensured that the cultural content of the adapted GSGL tool is acceptable for remote communities, whilst retaining acceptability in urban and regional communities. Multiple research team members in this study were previously involved in the development and validation studies for the urban/regional version of the GSGL tool. This has strengthened the methodology and contributed to a clear and thorough determination of equivalence between the original and adapted GSGL tool.

Some limitations were identified in the translation process of this study. Aboriginal and Torres Strait Islander languages are traditionally oral languages; translations were therefore completed using audio recordings. Whilst these methods are culturally relevant, some translation errors may be related to audio quality, or from not being able to easily refer to or confirm content of translations in writing. For example, although the Yumplatok words for ‘laugh’ and ‘life’ are phonetically different, the error in translation may have been due to audio recording issues. We mitigated the potential for translation errors through the study’s iterative translation process which enabled audio recording errors to be identified and rectified by multilingual expert committee members. This emphasises the importance of ensuring good quality audio recordings, and/or multiple audio recordings to reduce errors.

## Conclusion

The adapted GSGL tool is an acceptable and valid QoL tool for older remote-living Aboriginal and Torres Strait Islander peoples, and is valid across diverse regional, cultural and language groups. It is best practice to use an interpreter with the older person when assessment in another language is indicated. Further research will be completed to pilot the adapted GSGL tool in remote regions, and could potentially explore its applicability to other Indigenous populations internationally. Health and aged care services working with older Aboriginal and Torres Strait Islander peoples can use the adapted GSGL tool with confidence to assess QoL and to guide improvements to care and service delivery. Broader implementation of the adapted GSGL tool in aged care policy and practice is recommended to ensure ongoing assessment, care and evaluation of QoL for older Aboriginal and Torres Strait Islander peoples.

## Supplementary Information

Below is the link to the electronic supplementary material.

Supplementary Material 1

## Data Availability

Data Available Upon Request - The data supporting findings from this study are available from the corresponding author upon reasonable request. Due to confidentiality, the data are not publicly available.
