# Supplementary material for: Adaptation of the Good Spirit, Good Life quality of life tool for remote Indigenous Australians
Source: Qual Life Res. 2025 Oct 26;34(11):3245–55. doi: 10.1007/s11136-025-04083-x (PMC12681452; doi:10.1007/s11136-025-04083-x)
Supplement: Supplementary file 1 — Supplementary Material 1 [file 11136_2025_4083_MOESM1_ESM.docx]

**Appendix A.** Process for forward and back translation

**Stage 1. PREPARATION**

Explain the following process to translators for the study before commencing translation work.

Translators should be blinded to each other to keep each translation stages discrete and to ensure translators do not influence each other.

Equipment - audio recorder, pen/paper or computer to write out translations and feedback.

**It is important to record every step in the process. Write down any concerns or issues the translators have along the way. **

An additional person to assist with audio recording and writing notes will make the process easier. This is recommended but not essential.

**Stage 2. FIRST TRANSLATOR (forward translation)**

1. Preparing to translate: Familiarise yourself with the questions and consider how you would like to word these questions in Torres Strait Creole/Kimberley Kriol. For example, if there are some words that are not directly translatable and you will need to use other words/phrases as substitutions. E.g. can’t use yarn but can use talk. Knowing this in advance will help with a smoother translation.
2. Orally translate the questions from English to Torres Strait Creole/Kimberley Kriol using an audio recorder (i.e. Record the Torres Strait Creole/Kimberley Kriol version of the questions).
3. Listen to your translation to make sure it is correct.
4. Write out the translation as best as possible (the spelling doesn’t matter).
5. Write down any concerns or other feedback.

**Stage 3. SECOND TRANSLATOR (back translation)**

1. Listen to the audio recording from the first translator.
2. Orally translate the questions back to English from Torres Strait Creole/Kimberley Kriol. This should be audio recorded.
3. Listen to your back translation to make sure it is correct.
4. Write down the translation in English.
5. Write down any concerns or other feedback.

**Stage 4. REVIEW AND REFINE QUESTIONS**

1. Send written documents on forward and back translation stages to research team to review.
2. A follow up meeting will be scheduled with the research team to discuss any issues and possible ways of resolving these.

**Appendix B.** Iterative process with yarning groups and advisory groups

| **Original Item** | **Yarning groups** | | **Advisory groups** | | | | |
| --- | --- | --- | --- | --- | --- | --- | --- |
|  | **Issue raised** | **Suggestions for change** | **Kimberley** | | **Torres Strait** | **Perth** | |
| **Elder role**  Do you feel you can share your knowledge and stories with the younger mob? | The word ‘mob’ is not commonly used by older Torres Strait Islander people | Replace the word ‘mob’ with ‘generation’ or ‘people’ | Prefer ‘mob’ but ok with ‘people’ or ‘generation’ | | Prefer ‘people’ or ‘generation’ | Prefer ‘mob’ but ok with ‘people’ | |
|  |  |  | Agreement to change wording from ‘mob’ to ‘people’ | | | | |
| **Country/Island Home**  Do you feel you spend enough time connecting to Country? E.g. yarning about Country, going back to Country. | ‘Island Home’ is used instead of Country for Torres Strait Islander people | Adapt the question to include Island Home | Understood Island Home is preferred wording for Torres Strait Islander people | | Confirmed Island Home is preferred wording to Country | Understood Island Home is preferred wording for Torres Strait Islander people | |
|  |  |  | Agreement to include Island Home in wording and to present the question as two options. | | | | |
| **Community**  Do you feel connected to the Aboriginal and Torres Strait Islander community? | Change the wording from ‘the Aboriginal and Torres Strait Islander Community’ | Replace the wording with ‘your community’. | Preferred the wording ‘your community’ however agreed that ‘the Aboriginal and Torres Strait Islander community’ is broader | | Preferred the wording ‘your community’ however agreed that ‘the Aboriginal and Torres Strait Islander community’ is broader | | Preferred the original wording |
|  |  |  | Agreement to retain the original wording to be inclusive of all experiences of community. | | | | |
| **Culture**  Do you feel connected to cultural ways? E.g. attending Aboriginal events and meetings, sharing traditional foods. | Update and expand examples | Change ‘Aboriginal events and meetings’ to ‘cultural events and meetings’. Include painting, weaving, dancing and Sorry Time. | Agreement to update and expand examples. | | | | |
| **Health**  Do you do things to take care of your health? | Expand on the meaning of health | Include wording such as ’feeling good’, ‘feeling well’ or ‘wellbeing’. | Example of ‘mind, body and spirit’ proposed. | | | | |
|  |  |  | Example change not supported. | May need rewording. | | No alternative suggestion. | |
|  |  |  | Original item retained. | | | | |
| **Safety and Security**  Do you feel you have a safe place to live? | Experiences of racism reported. Safety and security issues are in the home and community. | Include the places where there are safety and security concerns | Prompt of ‘e.g. in your home/aged care home/community’ proposed. | | | | |
|  |  |  | Prompt endorsed by all advisory groups | | | | |
| **Supports and Services**  Do you feel the services you use are respectful and support your needs? | Need to identify which supports and services. | Name types of supports and services | Prompt of ‘e.g. your health/community/aged care services’ proposed. | | | | |
|  |  |  | Prompt endorsed by all advisory groups | | | | |
| **Respect**  Do you feel respected and valued as an Elder/older person? | Need to explain how respect is given | Include examples of respect from others | Example of “being listened to respectfully” proposed | | | | |
|  |  |  | Example endorsed by all advisory groups | | | | |
| **Future Planning**  Do you feel you have things in place as you grow older? E.g. your future health and care, funeral wishes, family looked after. | Broach topic with sensitivity. | Include examples of last wishes, advanced care plans, wills. Alternative wording of “do you have a plan in place for your future?” | Agreement on sensitivity of topic and need to navigate respectfully. Additional examples were considered to focus too heavily on end of life planning which is only one aspect of future planning. | | | | |
| **Basic Needs**  Do you feel you have enough money to get by? E.g. for food, housing, clothing. | Update and expand examples | Include examples such as fuel, bills, basic card. | Example update of ‘food, bills, transport, medication’ proposed. | | | | |
|  |  |  | Example update endorsed by advisory groups | | | | |
| **Family and friends**  Do you get to have a yarn and spend time with family or friends? | Original question endorsed by all yarning groups | | Original question endorsed by all advisory groups | | | | |
| **Spirituality**  Do you feel safe and supported in your spiritual beliefs? E.g. yarning about culture, going to church. | Original question endorsed by all yarning groups | | Original question endorsed by all advisory groups | | | | |

| 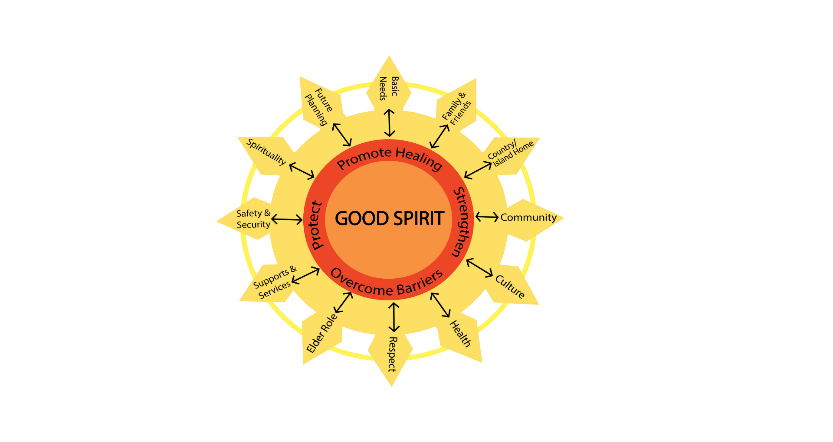 **Good Spirit Good Life Assessment (adapted version)** 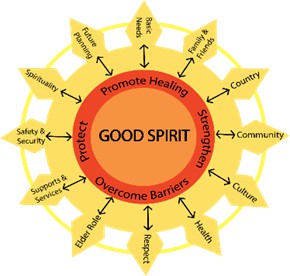 **Note to assessor**: Begin with social yarn to trust and rapport (see GSGL instruction booklet).  *Scoring*: If answer is *yes*, prompt further with  *all the time*, *most of the time* or *sometimes*.  If answer is no, prompt further with *not much*  or *never*.  Add detailed responses below each question to inform care.  **I would like to ask some questions on how you feel about your life today. There are no right or wrong answers.** | | 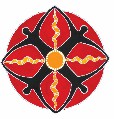 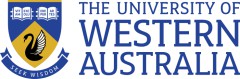  Tick most appropriate response | | | | |
| --- | --- | --- | --- | --- | --- | --- |
|  |  | YES | | | NO | |
|  |  | All the time (4) | Most of the time (3) | Sometimes (2) | Not much (1) | Never (0) |
| **1** | FAMILY AND FRIENDS  **Do you get to have a yarn and spend time with family or friends?** |  |  |  |  |  |
| **2** | COUNTRY/ISLAND HOME  ***For Aboriginal people:***  **Do you feel you spend enough time connecting to Country?**  Can prompt if needed e.g. yarning about Country, going back to Country  **or**  ***For Torres Strait Islander people:***  **Do you feel you spend enough time connecting to your Island Home?**  Can prompt if needed e.g. yarning about your Island Home, going back to your Island Home. |  |  |  |  |  |
| **3** | COMMUNITY  **Do you feel connected to the Aboriginal (and/or Torres Strait Islander) community?** |  |  |  |  |  |
| **4** | CULTURE  **Do you feel connected to cultural ways?**  Can prompt if needed e.g. through cultural events and meetings, traditional foods, activities such as painting, weaving, dancing, sorry time. |  |  |  |  |  |
| **5** | HEALTH  **Do you do things to take care of your health?** |  |  |  |  |  |

|  | | YES | | | NO | |
| --- | --- | --- | --- | --- | --- | --- |
|  |  | All the time (4) | Most of the time (3) | Sometimes (2) | Not much (1) | Never (0) |
| **6** | RESPECT  **Do you feel respected and valued as an Elder / older person?**  Can prompt if needed e.g. being listened to and spoken to respectfully |  |  |  |  |  |
| **7** | ELDER ROLE  **Do you feel you can share your knowledge and stories with the younger people?** |  |  |  |  |  |
| **8.** | SUPPORTS AND SERVICES  **Do you feel the services you use are respectful and support your needs?**  Can prompt if needed e.g. your health/community/aged care services.  **or**  *In residential care ask: **Do you feel this place is respectful and supports your needs?** |  |  |  |  |  |
| **9.** | SAFETY AND SECURITY  **Do you feel you have a safe place to live?**  Can prompt if needed e.g. in your home/aged care home/community. |  |  |  |  |  |
| **10.** | SPIRITUALITY  **Do you feel safe and supported in your spiritual beliefs?**  Can prompt if needed e.g. yarning about culture, going to church. |  |  |  |  |  |
| **11.** | FUTURE PLANNING  **Do you feel you have things in place as you grow older?**  Can prompt if needed e.g. your health care, housing, funeral wishes, family looked after. |  |  |  |  |  |
| **12.** | BASIC NEEDS  **Do you feel you have enough money to get by?**  Can prompt if needed e.g. for food, bills, transport, medication. |  |  |  |  |  |
| **TOTAL SCORE FOR EACH COLUMN** | |  |  |  |  |  |
| **OVERALL TOTAL SCORE /48** | |  | | | | |

**Scoring: An individual item score of 2 (sometimes) or below requires follow up**

All 12 items are **interconnected** and important to having a good life. When a person is not connected to one or more items (item score of 2 or below), this should be addressed.

See the **GSGL Recommendations Booklet** for strategies to strengthen connection and support quality of life
